# Better than expected: the influence of option expectations during decision-making

**DOI:** 10.1098/rspb.2018.2472

**Published:** 2018-12-12

**Authors:** Francesco Rigoli, Raymond Dolan

**Affiliations:** 1 Department of Psychology, City, University of London, Northampton Square, London EC1 V 0HB, UK; 2 The Wellcome Trust Centre for Neuroimaging, UCL, 12 Queen Square, London WC1N 3BG, UK; 3 Max Planck UCL Centre for Computational Psychiatry and Ageing Research, London WC1B 5EH, UK

**Keywords:** decision-making, option order, reference effects, risk

## Abstract

Our choices often arise from a consideration of options presented in a sequence (e.g. the products in a supermarket row). However, whether the precise sequential order of option presentation affects decision-making remains poorly understood. A recent model of choice proposes that, in a set of options presented sequentially, those that are better than expected will be perceived as more valuable, even when options are objectively equivalent within the set. Inspired by this proposal, we devised a novel decision-making task where we manipulated the order of option presentation together with expectations about option value. Even when we compared trials that were exactly equivalent except for option order, we observed a striking preference for options that were better than expected. Our findings show that expectations about options affect which option will be favoured within a sequence, an influence which is manifested as a preference for better-than-expected options. The findings have potential practical implications, as for example they may help policymakers in devising nudge strategies that rely on ad hoc option orders.

## 1. Background

Our decisions are rarely based on options that are presented simultaneously. For example, in supermarkets items are often placed at discrete locations and encountered one after the other. Therefore, an important research question is whether varying the order of option presentation influences the value of options, hence affecting decision-making. Scientists have investigated this question revealing a systematic preference for specific positions within a sequence of options [1–6]. Specifically, primacy effects, reflecting a preference for options offered early in a sequence, have emerged in some conditions while recency effects, corresponding to a preference for options offered later, have been observed in other conditions.

A recent model of choice [7,8] has raised the possibility that, independent of processes responsible for primacy and recency effects, another form of order effect could be critical when decision-making is characterized by sequential options. This influence would be exerted by expectations about the value of options. The model from which this proposal is derived [7,8] was developed to explain context effects on value attribution and choice. A central premise is that individuals keep track of the distribution of rewards encountered in an environment or context. When a stimulus is presented in a context, individuals are assumed to rely on knowledge about the contextual reward distribution when evaluating the stimulus. The model proposes that the subjective value of the stimulus is influenced by its associated reward prediction error (RPE). Specifically, the subjective value is predicted to increase if the stimulus is better than expected (relative to the contextual reward distribution), and to diminish if the stimulus is worse than expected.

This perspective has implications for scenarios where options are offered sequentially. Previous accounts have assumed that all options within a trial are equally affected by the context, even when presentation is sequential [7,8]. On the contrary, the model proposed by Rigoli *et al*. [7,8] implicates that options presented sequentially will not all be equally affected by context. Instead, each option will be influenced differently based on its associated RPE, namely based on the difference between the offered and expected reward for that option. Specifically, in an option sequence a greater (weaker) subjective value will be attributed to an option when it elicits a positive (negative) RPE. The prediction that, when options are offered sequentially, those that elicit a better RPE will be more attractive remains to be explored empirically, and here we provide such test. We designed a novel decision-making task where we varied both the order of option presentation and the expectations about option value. This allowed us to test the possibility that, when options are presented sequentially, those eliciting a positive (negative) RPE are favoured (disfavoured).

## 2. Methods

### (a) Participants

Forty-one healthy right-handed adults (23 females, 18 males; 18–40 years of age, mean age 24) participated in the study (data are available as the electronic supplementary material). Such sample size was selected prior to data collection to allow within-subjects statistical analyses aimed at testing a medium effect size (Cohen's *d* = 0.5) assuming a (two-tailed) significance threshold of 0.05 and a statistical power of 0.85. This requires 36 participants minimum. We decided to include five participants more than the minimum, resulting in 41 participants in total. All participants had normal or corrected-to-normal vision. None had a history of head injury, a diagnosis of any neurological or psychiatric condition, or was currently on medication affecting the central nervous system. The study was approved by the University College of London Research Ethics Committee. All participants provided written informed consent and were paid for participating. Participants were tested at the Wellcome Trust Centre for Neuroimaging at the University College London.

### (b) Experimental paradigm and procedure

**Figure 1.**
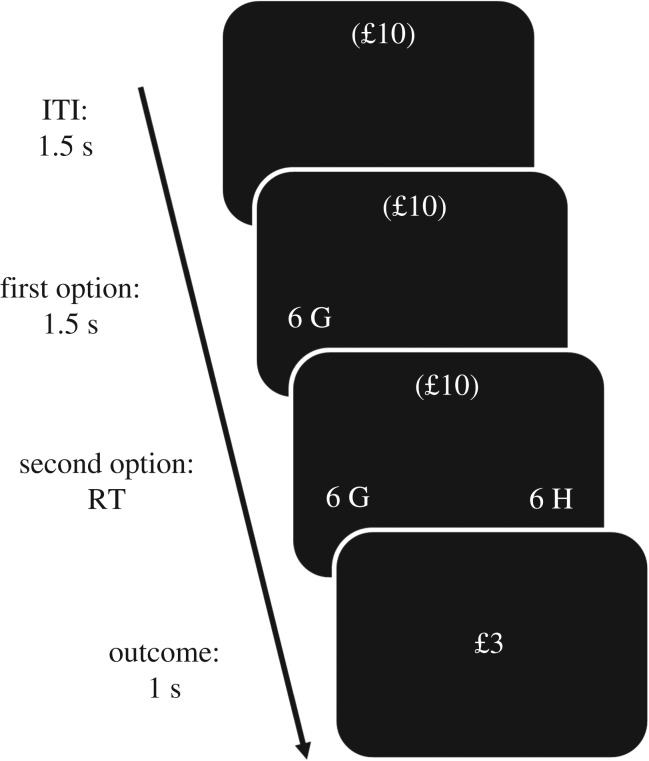
Task paradigm. On each trial, participants are presented with two options, one returning amount *x*/2 for sure (e.g. 6 H, where *x* = £6) and the other (e.g. 6 G) offering a 50/50 gamble between amount *x* and zero. Two different contexts alternate across blocks, namely a low-value context in which the amount *x* can be either £2 or £6 (resulting in the following choices: £1 versus £2/£0 and £3 versus £6/£0, respectively), and a high-value context in which the amount *x* can be either £6 or £10 (resulting in the following choices: £3 versus £6/£0 and £5 versus £10/£0, respectively). During an intertrial interval (ITI) of 1.5 s, participants are informed about the ongoing context by the corresponding amount in brackets (£10, as in this example, for the high-value context, and £2 for the low value context). Next, one option appears first (e.g. 6 G corresponding to the gamble), followed, after 1.5 s, by the second (e.g. 6 H corresponding to the sure option). The option order varies in such a way that the sure option appears first in half of the trials and the gamble in the other half. When the second option appears, choice can be realized (after a variable interval, depending on the response reaction time (RT)) and the outcome (e.g. £3) is revealed for 1 s.

Participants performed a computer-based task lasting approximately 25 min (figure 1). On each trial (200 trials were run in total), participants were offered two options, one associated with a sure gain of £ *x*/2 and the other with a 50/50 gamble between £ *x* or zero. Considering trials where *x* = £10 as an example, the sure option was displayed as ‘10 H’ (H indicates half of £10) and the gamble as ‘10 G’ (G indicates the gamble). Options were presented on the left and right sides of the screen, respectively (positions varied randomly). Two aspects were manipulated. First, options were presented sequentially, with the sure option appearing first for half of the trials and the gamble appearing first for the other half. Second, two different contexts alternated across blocks (each block comprised 20 trials; blocks alternated pseudo-randomly). In a low-value context, the amount *x* could be either £2 or £6 (resulting in the following choices: £1 versus £2/£0 and £3 versus £6/£0, respectively), while in a high-value context the amount *x* could be either £6 or £10 (resulting in the following choices: £3 versus £6/£0 and £5 versus £10/£0, respectively).

The ongoing context was explicitly signalled to participants throughout each block by a monetary amount shown in brackets on the top of the screen (£2 for the low-value context and £10 for the high-value context). Before a transition to a different context, a panel on the screen informed participants on the upcoming context. The first option appeared when a new trial started and was followed by the second option after 1.5 s. At this time point, one option could be selected by pressing either the left or the right arrow-key on the keyboard. Next, the outcome was revealed (appearing for 1 s), and was followed by an intertrial interval of 1.5 s. Participants had 3 s to finalize a choice. If a response occurred later, a ‘too late’ text appeared corresponding to a zero outcome.

Before the experiment, participants were instructed about the task, the gamble probability, and on how payment was derived. Then, they were familiarized with the task by performing 200 trials (this to reinforce their knowledge about context and the ensuing expectations about option value before the task started). At the end of the experiment, one single trial was randomly selected and the associated outcome was paid out in addition to a fixed £5 endowment.

### (c) Predictions

Predictions derived from Rigoli *et al*. [7,8] in this task can be examined by focusing on the low-value context first. During trials in which *x* = £6, the option presented first informs participants that this is a relatively good trial (given that the alternative condition in the low-value context has *x* = £2), and therefore presentation of this first option elicits a positive RPE. Note that, by design, in our task presentation of the second option never provides any extra information, and consequently it is always associated with a null RPE. By contrast, considering the high-value context, during trials in which *x* = £6 the option presented first informs participants that this is a relatively bad trial (given that the alternative condition in the high-value context has *x* = £10), and therefore presentation of the first option elicits a negative RPE. Hence, the model of Rigoli *et al*. [7,8] predicts that, when *x* = £6, the option presented first should be preferred more in the low- compared to the high-value context. Following an analogous line of reasoning, the option presented first is assumed to elicit a negative RPE when *x* = £2 and a positive RPE when *x* = £10, hence predicting that it should be selected more often in the latter compared to the former condition.

**Figure 2.**
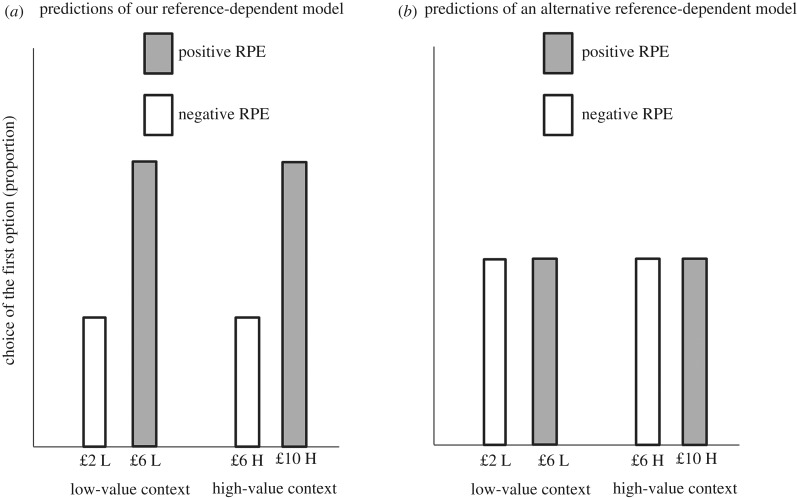
Model's prediction. (*a*) Predicted proportion of the choice of the first option for the different conditions (L = low-value context; H = high-value context), derived from the reference-dependent model of Rigoli *et al*. [7,8]. (*b*) Predicted proportion of the choice of the first option for the different conditions, derived from an alternative reference-dependent model where the context influences equally all options within a sequence.

The same reasoning can be expressed formally. The model of Rigoli *et al*. [7,8] proposes that, in a sequence of options, the subjective value *V*(*y*) of an option associated with position *y* and reward *R*(*y*) corresponds to a weighted RPE (adapted from [7,8]):

2.1 $$V(y)=\frac{\left(R(y)-E(y)\right)}{\sigma^{2}},$$

where *E*(*y*) is the expected reward (which depends on expectations elicited by the context) for the option associated with position *y*, and *σ*^2^ is an uncertainty parameter (here assumed to be constant for all options). Crucially, the model proposes that the expected reward *E*(*y*) is a function of the position of an option within a sequence, implying that it can vary across options within the same trial. We can use the model to simulate choice behaviour in our task (figure 2*a*). This simulation is based on two assumptions which reflect the task structure. First, to capture the manipulation of context, we assumed that the expected reward associated with the option presented first during the low-value context *E*_L_(1) is lower than the expected reward associated with the option presented first during the high-value context *E*_H_(1) (i.e. *E*_L_(1) < *E*_H_(1)). This implies that, according to equation (2.1), the subjective value of the option presented first will increase and decrease in the low-value and high-value context, respectively. Second, to capture the fact that contextual expectations do not affect the option presented second, we assumed that, for all conditions, the expected reward for the option presented second *E_i_*(2) is equal to the reward associated with the option presented first *R_i_*(1) (i.e. *E_i_*(2) = *R_i_*(1)). This implies that, according to equation (2.1), the subjective value of the option presented second does not vary across conditions. Figure 2*a* shows that, according to this model, the predicted proportion of choices of the option presented first will be higher for £10 in the high-value context and for £6 in the low-value context. We can compare these predictions against an alternative reference model (figure 2*b*) where expectations elicited by the context affect both available options equally, independent of their position (in other words where *E*_L_(*y*) > *E*_H_(*y*) and *E_i_*(1) = *E_i_*(2)). This alternative reference model does not predict any order effect, as the proportion of choice of the option presented first is equal for all conditions (figure 2*b*).

The model of Rigoli *et al*. [7,8] raises the specific prediction that a preference for the option presented first will vary across conditions as shown in figure 2*a*. The model is agnostic about whether a primacy effect (i.e. an overall preference for the first option) or recency effect (an overall preference for the second option) will also emerge. These effects have been reported previously [1–6] and could also be assessed in our task. However, we emphasize that our task was optimized to investigate predictions derived from Rigoli *et al.* [7,8] and not for examining recency and primacy effects (see Discussion for a comparison between our task and prior literature on recency and primacy effects).

## 3. Results

We first characterized a general gambling disposition in our task (table 1; figure 2). The total proportion of gamble choices did not differ significantly from 0.5 (table 1; figure 2; mean = 0.43; median = 0.53; s.d. = 0.26; *t*_40_ = −1.66, *p* = 0.105; *d* = −0.26; two-tailed alpha < 0.05 was used as significance criterion in all analyses). In other words, although individual differences were evident with respect to the attractiveness of the gamble (table 1; figure 2), participants on average were neither risk-seeking nor risk-averse. The observation of an absence of risk aversion during decisions involving gains is inconsistent with prevailing theories (e.g. [9,10]), though it replicates previous research using analogous tasks [7,11,12]. This pattern can be explained by the possibility that an increased risk propensity occurs when small monetary amounts are at stake [13].

**Table 1.** For different conditions, the table reports descriptive statistics regarding to the proportion of choice of the gamble (gambling first: the gamble is presented as first option; gambling second: the gamble is presented as second option; L = low-value context; H = high-value context).

condition	mean	95% CI	s.d.	median	min	max
total	0.43	[0.35–0.52]	0.26	0.53	0	0.85
positive RPE; gamble first	0.45	[0.37–0.54]	0.27	0.48	0	1
negative RPE; gamble first	0.41	[0.32–0.52]	0.31	0.46	0	1
positive RPE; gamble second	0.41	[0.32–0.49]	0.27	0.50	0	1
negative RPE; gamble second	0.45	[0.36–0.55]	0.30	0.52	0	1
£2 L; gamble first	0.43	[0.33–0.54]	0.33	0.48	0	1
£6 L; gamble first	0.45	[0.35–0.56]	0.33	0.48	0	1
£6 H; gamble first	0.41	[0.30–0.51]	0.33	0.40	0	1
£10 H; gamble first	0.45	[0.36–0.55]	0.29	0.48	0	1
£2 L; gamble second	0.46	[0.35–0.57]	0.34	0.48	0	1
£6 L; gamble second	0.41	[0.31–0.51]	0.32	0.40	0	1
£6 H; gamble second	0.45	[0.35–0.55]	0.32	0.52	0	1
£10 H; gamble second	0.40	[0.31–0.49]	0.29	0.44	0	1

**Figure 3.**
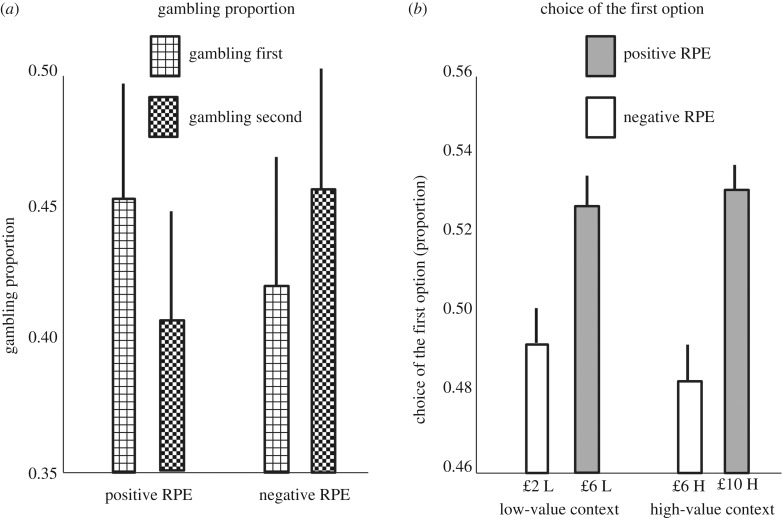
Empirical data. (*a*) The proportion of gambling choices (±s.e.) is shown considering RPE and CFO (gambling first: the gamble is presented as first option; gambling second: the gamble is presented as second option) as factors. A significant RPE-CFO interaction emerged from these data (*F*_1,40_ = 15.43, *p* < 0.001; $\eta_{P}^{2}=0.278$). (*b*) The proportion of choices of the option presented first (±s.e.) is shown considering RPE and context (L = low-value context; H = high-value context) as factors. A main effect of RPE emerges from these data (*F*_1,40_ = 10.00, *p* = 0.003; $\eta_{P}^{2}=0.200$), but no RPE-context interaction (*F*_1,40_ = 0.17, *p* = 0.684; $\eta_{P}^{2}=0.004$).

Next, we asked whether gambling varied across the different conditions. To test this, we ran a 2 × 2 × 2 ANOVA of gambling proportion with context (high-value versus low-value), RPE (positive versus negative) and category of the first option (CFO; reflecting whether the option presented first was the gamble or the sure option) as factors (table 1; figure 3*a*). Specifically, with respect to the context and RPE factors, trials where *x* = £2 were associated with low-value context and negative RPE; trials where *x* = £10 were associated with high-value context and positive RPE; trials where *x* = £6 were associated either with low-value context and positive RPE or with high-value context and negative RPE. Results showed no main effect of context (*F*_1,40_ = 0.17, *p* = 0.677; $\eta_{P}^{2}=0.004$), no main effect of RPE (*F*_1,40_ = 0.05, *p* = 0.831; $\eta_{P}^{2}\;=0.001$), no main effect of CFO (*F*_1,40_ = 0.14, *p* = 0.715; $\eta_{P}^{2}=0.003$), no context-RPE interaction (*F*_1,40_ = 0.04, *p* = 0.847; $\eta_{P}^{2}\;=0.001$), no context-CFO interaction (*F*_1,40_ = 0.03, *p* = 0.875; $\eta_{P}^{2}\;=0.001$), and no three-way interaction (*F*_1,40_ = 0.40, *p* = 0.529; $\eta_{P}^{2}=0.010$). However, we found a significant RPE-CFO interaction (*F*_1,40_ = 15.43, *p* < 0.001; $\eta_{P}^{2}\;=0.278$). This effect indicates that, comparing trials where the first option elicits a positive RPE against trials where it elicits a negative RPE, gambling increases when the first option is a gamble (figure 3*a*). This finding is consistent with our prediction derived from Rigoli *et al*. [7,8], because it shows that, when presentation of the gamble elicits a positive RPE, gambling increases.

Previous research on decision-making conditions where options are presented sequentially has asked whether any position in a sequence systematically boosts preference [1–6]. Primacy effects (i.e. a preference for options presented first) have been observed, though in some conditions recency effects (i.e. a preference for options presented last) also arise [5]. Although our task was not optimized for investigating these effects, we asked whether primacy or recency effects also emerged from our data. We found that the total proportion of choices for the option presented first did not differ significantly from 0.5 (table 2; figure 3*b*; mean = 0.50; median = 0.50; s.d. = 0.05; *t*_40_ = 0.38, *p* = 0.708; *d* = 0.06). In other words, participants did not exhibit any systematic preference for the first nor for the second option in the sequence.

**Table 2.** For different conditions, the table reports descriptive statistics relative to the proportion of choice of the option presented first (gambling first: the gamble is presented as first option; gambling second: the gamble is presented as second option; L = low-value context; H = high-value context).

condition	mean	95% CI	s.d.	median	min	max
total	0.50	[0.49–0.52]	0.054	0.50	0.38	0.72
positive RPE	0.48	[0.46–0.50]	0.065	0.49	0.31	0.72
negative RPE	0.52	[0.50–0.54]	0.061	0.51	0.44	0.72
£2 L; gamble first	0.43	[0.33–0.54]	0.33	0.48	0	1
£6 L; gamble first	0.45	[0.35–0.56]	0.33	0.48	0	1
£6 H; gamble first	0.41	[0.30–0.51]	0.33	0.40	0	1
£10 H; gamble first	0.45	[0.36–0.55]	0.29	0.48	0	1
£2 L; gamble second	0.54	[0.43–0.65]	0.34	0.52	0	1
£6 L; gamble second	0.59	[0.48–0.69]	0.32	0.60	0	1
£6 H; gamble second	0.55	[0.45–0.65]	0.32	0.48	0	1
£10 H; gamble second	0.60	[0.51–0.69]	0.29	0.56	0	1

Next, we examined the specific prediction of the model of Rigoli *et al*. [7,8] with respect to the probability of choosing the option presented first for the different conditions, which is described in figure 2*a*. To test this, we ran a 2 × 2 × 2 ANOVA as above (having context, RPE and CFO as factors) but where the dependent variable was now the proportion of choices for the first option (table 2; figure 3*b*). This analysis showed no main effect of context (*F*_1,40_ = 0.17, *p* = 0.684; $\eta_{P}^{2}=0.004$), no main effect of CFO (*F*_1,40_ = 2.75, *p* = 0.105; $\eta_{P}^{2}=0.065$), no context-RPE interaction (*F*_1,40_ = 0.17, *p* = 0.684; $\eta_{P}^{2}=0.004$), no context-CFO interaction (*F*_1,40_ = 0.17, *p* = 0.678; $\eta_{P}^{2}=0.004$), no CFO-RPE interaction (*F*_1,40_ = 0.05, *p* = 0.832; $\eta_{P}^{2}=0.001$), and no three-way interaction (*F*_1,40_ = 0.04, *p* = 0.847; $\eta_{P}^{2}=0.001$). However, we observed a main effect of RPE (*F*_1,40_ = 10.00, *p* = 0.003; $\eta_{P}^{2}=0.200$). Consistent with our prediction [7,8], this effect is driven by a larger proportion of choices for the option presented first when the RPE is positive compared to negative. The results of this analysis fit with model's predictions described in figure 2*a*.

In our task, trials where *x* = £6 were exactly equivalent across contexts, except that in the low-value context presentation of the first option elicited a positive RPE, while in the high-value context it elicited a negative RPE. Therefore, the most stringent test of our hypotheses is a comparison between low- and high-value context for trials where *x* = £6. Consistent with our prediction [7,8], when comparing low- and high-value context for trials where *x* = £6, our analyses revealed a higher proportion of choices for the option presented first (table 2; figure 3*b*; *t*_40_ = 3.54, *p* = 0.001; *d_z_* = 0.55).

In short, we found that our task was characterized by an absence of systematic risk seeking or risk aversion, that gambling preference was unaffected by the different task conditions, and we observed no primacy or recency effects induced by a sequential presentation of options. Nevertheless, consistent with hypotheses derived from a previous model [7,8], our analyses revealed an increased likelihood of selecting the option presented first when that option signalled a better-than-expected value, compared to when it signalled a value that was worse than expected.

## 4. Discussion

We studied the impact of presenting options sequentially on decision-making. By manipulating a contextual reward distribution, we induced different expectations so that the option presented first in a sequence elicited either a positive or negative RPE. We found that the first option was favoured when it elicited a positive, compared to a negative, RPE. To our knowledge, this represents the first evidence of documenting an effect of option order exerted by expectations about option value.

Our observations fit with theories viewing subjective value as inherently context-dependent [7,8,11,12,14–28]. According to this perspective, the subjective value of a stimulus derives from a comparison of the stimulus against other potential stimuli in a context. Our study contributes to this literature by supporting the notion that, when options are offered sequentially, context does not affect all options equally, but it can induce specific expectations for different positions and hence affect each option differently. This process is consistent with the form of order effect observed here, whereby the context determines whether a given position (in our study, the first position of the sequence) will be favoured or not.

While previous research has documented primacy and recency effects emerging when options are presented sequentially during decision-making [1–6], these effects were absent in our task. This raises the question of which conditions allow primacy and recency effects to emerge. To answer this, it is helpful to consider the key differences between prior literature and our task design. In the former, participants were presented with novel options (i.e. options never experienced before) and the attributes of an option had to be learnt at the time when the option was on offer. For example, in a study asking participants to taste and evaluate a new wine, the wine's features were learnt while the wine was offered as an option [5]. This focus on learning is absent in our study, where participants were already familiar with the options (given the long training session). Another difference is that previous literature adopted longer temporal delays between subsequent options (e.g. [5]) and presented each option for a longer time (e.g. [4,5]). Some studies also included a filler task between option presentation and choice [4]. These features, charactering previous research but absent in our task, are likely to engage memory processes that could be critical for primacy and recency effects. Finally, in previous literature options were characterized by many and relatively complex attributes (e.g. in the study involving decision-making about wine; [5]). On the contrary, our study relied upon simple choices involving monetary amounts and probabilities. Altogether, the focus on learning, memory and on complex options, characterizing previous literature but not our study, could explain why recency and primacy effects were absent in our data.

A critical variable in tasks where options are offered sequentially is the temporal interval characterizing option presentation. It remains unknown whether the delay between options modulates order effects, a question which remains open with respect to classical primacy and recency effects as well as with respect to the order effects highlighted here. A related question is whether order effects vary when comparing conditions engaging memory (i.e. when an option disappears after it is presented, although it remains available; as in previous research on recency and primacy effects) against conditions where memory is not engaged (when an option does not disappear after it is presented; as in this study).

Our observations are relevant to a number of practical domains. The notion of a nudge is used to characterize decision frames designed by policymakers to safeguard choice freedom and simultaneously promote common goals [29]. Our findings may help in devising effective nudges, by suggesting a benefit from locating a target option in temporal and spatial positions where it is appraised as better than expected.

## Supplementary Material

Data

## Data accessibility

Empirical data of the study are available as the electronic supplementary material.
